# Supplementary material for: Empirical methods for controlling false positives and estimating confidence in ChIP-Seq peaks
Source: BMC Bioinformatics. 2008 Dec 5;9:523. doi: 10.1186/1471-2105-9-523 (PMC2628906; doi:10.1186/1471-2105-9-523)
Supplement: Additional File 1 — A variety of html documents from the USeq web site detailing the available applications, their best usage, output file type descriptions, command line menus, etc. [file 1471-2105-9-523-S1.zip › USeqUserGuides/JavaDoc.zip!/JavaDoc/util/gen/package-tree.html]

util.gen Class Hierarchy


|  |  |  |
| --- | --- | --- |
| **Overview** \| **Package** \| Class \| Use \| **Tree** \| **Deprecated** \| **Index** \| **Help** | |  |
| **PREV**   **NEXT** | **FRAMES**    **NO FRAMES**     **All Classes** |  |


---


## Hierarchy For Package util.gen

**Package Hierarchies:**: All Packages

---

## Class Hierarchy

- java.lang.Object
  - util.gen.**Bin**- java.awt.Component (implements java.awt.image.ImageObserver, java.awt.MenuContainer, java.io.Serializable)
      - java.awt.Container
        - java.awt.Window (implements javax.accessibility.Accessible)
          - java.awt.Frame (implements java.awt.MenuContainer)
            - javax.swing.JFrame (implements javax.accessibility.Accessible, javax.swing.RootPaneContainer, javax.swing.WindowConstants)
              - util.gen.**TextFrame**
      - util.gen.**CubicSpline**- util.gen.**Email**- util.gen.**FileSize** (implements java.lang.Comparable<T>)- util.gen.**Histogram**- util.gen.**HTML**- util.gen.**IO**- util.gen.**Job**- util.gen.**LinearRegression**- util.gen.**MD5Crypt**- java.security.MessageDigestSpi
                          - java.security.MessageDigest
                            - util.gen.**MD5** (implements java.lang.Cloneable)
                          - util.gen.**Misc**- util.gen.**Num**- util.gen.**Passwords**- util.gen.**PearsonCorrelation**- util.gen.**RankedFloatArray**- util.gen.**RankSample**- util.gen.**RankSampleIndexComparator** (implements java.util.Comparator<T>)- util.gen.**RankSampleValueComparator** (implements java.util.Comparator<T>)- util.gen.**ReadZip**- util.gen.**SpearmanCorrelation**- util.gen.**SQL**- util.gen.**Swing**- util.gen.**XML**

---


|  |  |  |
| --- | --- | --- |
| **Overview** \| **Package** \| Class \| Use \| **Tree** \| **Deprecated** \| **Index** \| **Help** | |  |
| **PREV**   **NEXT** | **FRAMES**    **NO FRAMES**     **All Classes** |  |


---
